# Impact of primary glaucoma on health-related quality of life in China: the handan eye study

**DOI:** 10.1186/s12886-023-03106-w

**Published:** 2023-09-14

**Authors:** Di Song, Sujie Fan, Qiang Zhou, Xiaohui Yang, Sizhen Li, Lynne Lohfeld, Weihe Zhou, Nathan Congdon, Yuanbo Liang, Ningli Wang

**Affiliations:** 1 National Clinical Research Center for Eye Diseases, The Eye Hospital, School of Optometry and Ophthalmology, Wenzhou Medical University, Wenzhou, Zhejiang China; 2 The First People’s Hospital of Huzhou, The First Affiliated Hospital of Huzhou Teacher College, Huzhou, Zhejiang, China; 3 Handan Eye Hospital, Handan, Hebei, China; 4 Beijing Chaoyang Hospital, Beijing, China; 5 Beijing Tongren Hospital, Capital Medical University, Beijing, China; 6 Department of Ophthalmology and Visual Sciences, The Chinese University of Hong Kong, Hong Kong, China; 7 Centre for Public Health, Queen’s University, Belfast, UK; 8 Glaucoma Institute, Wenzhou Medical University, Wenzhou, Zhejiang China; 9 Orbis International, New York, NY USA

**Keywords:** Glaucoma, EQ-5D, Health-Related Quality of Life, Visual function quality of life

## Abstract

**Background:**

We assessed health-related quality of life (HRQOL) and its determinants among rural glaucoma participants compared to age-matched normal controls in the population-based Handan Eye Study (HES), in rural Yongnian County, northern China.

**Methods:**

We enrolled 99 adults with glaucoma (mean age 63.0 ± 11.0 years), including primary open-angle glaucoma (POAG, n = 67) and primary angle-closure glaucoma (PACG, n = 32) and 102 controls (mean age 58.5 ± 5.3 years) with normal visual acuity and visual field and no history of glaucoma. Results of ophthalmic examinations and socioeconomic data were recorded. HRQOL was measured using the EQ-5D (converted to utility valves, UVs), and visual function (VF) and vision-related quality of life (VRQOL) were evaluated using the visual function-quality of life (VF-QOL) instrument.

**Primary and secondary outcome measures:**

EQ-5D and VF-QOL scores.

**Results:**

The mean UVs, VF, and VRQOL scores for glaucoma cases were 0.98 ± 0.04, 87.9 ± 15.2, and 95.5 ± 12.8, respectively, significantly worse than VF (94.4 ± 4.4) and VRQOL (100.0 ± 0.0) among controls, even after adjusting for age, gender, educational level, and family income (*P* = 0.015, *P* = 0.033). UVs were significantly lower among glaucoma participants with impaired VRQOL (55.4 ± 11.5) compared to those with normal VRQOL scores (99.1 ± 2.8) (UVs: 0.92 ± 0.08 vs. 0.99 ± 0.03, *P* = 0.036), also after adjustment for age and family income (*P* = 0.006). Participants with PACG had significantly lower VF and VRQOL scores compared to POAG (77.8 ± 21.4 vs. 92.9 ± 6.8, P < 0.001; 89.0 ± 18.1 vs. 98.7 ± 7.5, P < 0.001).

**Conclusion:**

Participants with glaucoma have worse visual function and related quality of life compared to age-matched normal population controls. Participants with PACG have lower VF and VRQOL compared to those with POAG. UVs can be used for cost-effectiveness research and to support public health strategies for glaucoma in rural China.

**Supplementary Information:**

The online version contains supplementary material available at 10.1186/s12886-023-03106-w.

## Background

Glaucoma is the world’s second-leading cause of blindness, and by 2040 will affect 111.8 million people globally between the ages of 40 and 80, with Asia accounting for 60% of cases [1]. In China, primary open-angle glaucoma (POAG) affects 1.0% [2] of rural adults aged 30 years and above, and primary angle-closure glaucoma (PACG) approximately 0.5% [3, 4].

Glaucoma represents a significant public health threat, and its burden on society is increasing with population aging. The annual costs for initial treatment of PACG and POAG are approximately US$600 and US$345, rising to $8920 and $3600 annually, respectively, for bilateral blindness [5]. This may be compared to the per-capita gross domestic product (GDP) for China’s rural and urban regions of $4010 and $10,800, respectively [5].

Characteristics of glaucoma may include irreversible optic nerve damage and gradual onset of visual field defects, even to the point of central blindness. Glaucoma negatively affects visual function (VF) and vision-related quality of life (VRQOL), and may be associated with anxiety and depression [6] and poor quality of life (QOL) [7].

The visual function-quality of life (VF-QOL) [8] instrument can be used to estimate the VF and VRQOL scores for cataract patients, yielding high efficacy. Although the quality of life of glaucoma patients has been extensively studied, existing publications are all clinic-based, and thus give no idea of the QOL impact of glaucoma on the majority of rural persons with undiagnosed disease. The glaucoma participants included the current study were part of the Handan Eye Study (HES), a population-based survey of eye diseases in rural China, providing for the first time a truly representative sample of affected and unaffected persons.

Although VF-QOL can be used to demonstrate the impact of glaucoma on patients encountered during clinical practice, these data cannot be converted to utility values (UVs), [9] which can in turn be used to calculate crucial cost-effectiveness figures. To optimize the allocation of limited resources for health care interventions, cost-effectiveness analysis (CEA) has been increasingly used to improve decision-making. UVs can also be used to measure Health-related quality of life (HRQOL), and to calculate Quality Adjusted Life Years (QALYs) [10, 11], a common outcome allowing comparisons between diseases and clinical outcomes.

EQ-5D is a widely used tool for measuring UVs [12, 13], recommended by the United Kingdom National Health Institute for Clinical Excellence (NICE) [14]. To our knowledge, no population-based studies have previously reported UVs derived from EQ-5D among persons with glaucoma in China. Thus, we sought to assess HRQOL and its determinants using EQ-5D and VF and VRQOL scores using the VF-QOL instrument for a population-based sample of glaucoma participants, compared to population-based, age-matched normal controls.

## Materials and methods

The cohort in this article was enrolled from the Handan Eye Study (HES), which included a total of 99 participants with primary glaucoma. Because there are no population-based studies on EQ-5D values representing primary glaucoma in rural China compared to the normal population, we selected normal controls from the Handan Eye Survey for comparison. Thus, this is a population-based, nested, cross-sectional case-control study. The HES is the largest comprehensive prevalence survey of eye disease carried out in a rural population in China, designed to examine the prevalence of blindness and visual impairment, risk factors for ocular disease, and barriers to accessing eye care services among community-dwelling persons aged 30 years and above. Details of the study design and data collection have been described previously [15].

Briefly, 13 villages were randomly selected using a clustered sampling technique, with the size of each cluster proportional to the population size. People who resided for at least 6 months in the target villages were invited for a detailed eye examination at a designated examination site (Yongnian County Hospital), with a brief home examination offered to those who could not attend. The demographic and socioeconomic characteristics of participants were collected by questionnaire interview during the examination. A screening process was also conducted on the names listed on the census roles for selected populations, with 8653 names screened and 7557 individuals ultimately confirmed as eligible. All study procedures adhered to the principles outlined in the Declaration of Helsinki for research involving human subjects. Ethics approval was obtained from the Beijing Tongren Hospital review board, and written informed consent was obtained from all participants.

### Visual field evaluation

The HES carried out 24 − 2 Swedish Interactive Testing Algorithm (SITA) visual field testing, performed using the Humphrey Visual Field Analyzer (Carl Zeiss, Jena, Germany) on a 10% random smaple of non-glaucoma participants. In addition, all participants with angle-closure glaucoma on gonioscopy and/or suspected glaucoma underwent SITA standard visual field testing. Tests were repeated the glaucoma hemifield test was outside normal limits or borderline or if the test was unreliable) [3]. According to the International Glaucoma Association, glaucoma was defined as a hemifield test result outside normal limits combined with a cluster of four or more contiguous points on the pattern deviation plot (P < 0.05%) not crossing the horizontal meridian [4, 16]. Visual field loss (VFL) was categorized into mild, moderate, and severe stages based on the Hodapp classification system [17].

### Assessment of visual acuity (VA) and automated refraction

The presenting visual acuity (PVA) of participants, wearing spectacles if available, was tested monocularly (right followed by left eye) and binocularly using a Logarithmic Vision Chart (Precision Vision, La Salle, IL, USA) at 4 m [15]. Measurements for monocular tests were taken by occluding the contralateral eye with an eye patch. More details can be found in the published HES protocol [15].

### Definitions of glaucoma and normal participants

Three senior glaucoma specialists reviewed visual field and optic nerve photographs and clinical records for vertical cup-to-disc ratios, categorizing participants as having definite, probable, possible, or no glaucoma based on consensus. A more detailed description of the methodology for determining glaucoma status and diagnosis has been reported elsewhere [2, 3]. “Primary glaucoma” includes only definite cases of primary open-angle and primary angle-closure glaucoma, which was the main cause of lower visual acuity and visual field defect. All suspected glaucoma participants underwent visual field testing. The definition of older age was ≥ 61 years and lower income was defined as ≤ $ 507 the local mean per capita income.

Participants were considered normal (non-glaucomatous) based on the following criteria: having completed all examinations, questionnaires, EQ-5D, VF-QOL, a reliable visual field test; no visual field loss (Mean Defect > -2 dB) and presenting visual acuity of 6/12 or better in the worse eye [7]. To balance, the age range of the controls and cases, the following steps were used: a. Maintain the cases sample as it is (sample size of 99); b, Calculate the first and third quartiles of the age within the cases sample;c,Draw randomly, from the overall controls, a sample of size 102, such that the age is between the first and third quartiles of the age within the cases sample; the generated sample of size 102 is used as the control sample. This process enables the cases and the controls to have similar age profile and minimises the difference of the average age between the two samplesCriteria for exclusion of glaucoma cases or Controls from the study included absent data on the EQ-5D or VF-QOL instruments, though such persons were included in all analyses for items on these tests for which they provided data.

### Utility values and visual functioning quality of life

Trained interviewers conducted interviews with study participants, collecting data on various factors, including demographic information, quality of life assessments using the EQ-5D and Visual Functioning-Quality of Life (VF-QoL) instruments [8, 18], co-morbidities [19], family history of eye disease, and any barriers that prevented the participants from seeking eye care [15].

The EQ-5D instrument consists [20] of five dimensions: mobility, self-care, usual activities, pain or discomfort, and anxiety or depression. Each dimension describes a participant’s health status at 1 of 3 severity levels: 1-no problems, 2-moderate problems, and 3-extreme problems. The participant’s responses were converted to UVs based on the Chinese value set [9, 20].

The Chinese version of the VF-QOL questionnaire [8] includes one general vision question and 12 additional questions divided into four subscales: visual perception (activity limitation, near vision, intermediate vision, distance vision); sensory adaptation (light/dark adaptation, visual search, color discrimination, glare disability); peripheral vision (one question); and depth perception (one question). Four questions were related to activities of daily living: self-care (bathing, eating, dressing, toileting); mobility (walking to neighbors, walking to shops, doing household chores); social interaction (attending functions, meeting with friends); and mental well-being (burden on others, dejection, loss of confidence). Participants responded to each question regarding the difficulty level, from “not at all” to “a lot”. These responses were linearly transformed to a total possible score ranging from zero (representing maximum difficulty) to 100 (representing no problems). To account for the impact of subjective visual function on utility values, we grouped the participants according to the VF-QOL results. Zhao et al. [8] reported that the mean VF and QOL scores for normal people were 83.8 and 90.2, respectively. According to the mean VF score, the glaucoma participants were divided into a normal vision group (VF score above 83.8) and an impaired vision group (VF score below 83.8). Based on the mean QOL score, the glaucoma participants were divided into normal vision (mean QOL score above 90.2) and impaired vision (mean QOL score below 90.2) groups.

### Statistical analyses

Data analysis was performed with SPSS 20.0 for Windows (SPSS, Inc., Chicago, IL). The mean and standard deviation of UVs on the EQ-5D and scores on the VF and VRQOL scores were calculated. The means, SDs, and 95% CIs were calculated for the UVs, VF, and QOL scores. Sociodemographic and clinical characteristics were compared between participants with and without primary glaucoma using the χ^2^ test for categorical variables and the *Student’s t-test* for continuous variables. One-way ANOVA post-comparison followed by pairwise comparison by Bonferroni correction was used to compare the mean defect (MD) in visual field testing between mild, moderate, and severe glaucoma groups. The UVs, VF, and VRQOL scores across different sociodemographic and clinical characteristics were compared between subgroups of glaucoma using the *Student’s t-test* and* χ*^*2*^*tests* for categorical variables. Multiple linear regression was performed for factors significantly associated with UVs, VF, and QOL scores. Spearman correlation coefficients were used to quantify the association between UVs and VF/QOL scores. Two-sided *P-values* < 0.05 were statistically significant.

## Results

### Sociodemographic and clinical characteristics

A total of 99 cases of primary glaucoma were identified, including POAG (n = 67, 67.7%) and PACG (n = 32, 32.3%) (Table 1). Altogether, 102 normal controls were also analyzed. Participants with primary glaucoma exhibited a female predominance (n = 61, 61.6%), and a mean age of 63.0 ± 11.0 years. Table 1 displays the characteristics of study participants with respect to education, household income, participation in rural New Cooperative Medical Service health insurance, and presence of comorbidities.

**Table 1:** Sociodemographic and Clinical Characteristics of 99 participants with primary glaucoma and 102 without, values represent number (%) unless otherwise stated

Characteristic	Primary Glaucoma (n = 99)	Normal Controls (n = 102)	*p*
Age, y (mean ± SD)	63.0 ± 11.0	58.5 ± 5.3	< 0.001
Female Gender	61 (61.6)	44 (43.1)	**0.013**
Primary education or above	65 (65.7)	90 (88.2)	< 0.001
Family income below the median for the area	31 (31.3)*	51 (50.0)**	**0.003**
Participate in rural New Cooperative Medical Service health insurance	61 (61.6)	69 (67.6)	0.455
Comorbidity present †	53(53.5)	57 (55.9)	0.847
EQ-5D	0.982 ± 0.042	0.991 ± 0.025	0.092
VF total	87.9 ± 15.2	94.4 ± 4.4	**< 0.001**
QOL total	95.5 ± 12.8	100 ± 0.0	**0.001**
VF-scale 1	86.1 ± 17.3	94.8 ± 7.5	**< 0.001**
VF-scale 2	90.4 ± 20.4	97.6 ± 9.9	**0.002**
VF-scale 3	79.8.±14.1	85.6 ± 6.4	**< 0.001**
VF-scale 4	94.8 ± 16.2	99.7 ± 3.4	**0.005**
QOL-scale 1	97.3 ± 9.5	100 ± 0.0	**0.005**
QOL-scale 2	95.3 ± 15.5	100 ± 0.0	**0.004**
QOL-scale 3	93.3 ± 19.0	100 ± 0.0	**0.001**
QOL-scale 4	96.1 ± 14.7	100 ± 0.	**0.011**

*Data missing for 28 persons **Data missing for 29 persons

† Comorbidity present: Self-reported comorbidities include diabetes, arthritis, stroke/brain hemorrhage, high blood pressure, angina, heart attack, heart failure, and asthma

ƪ Adjusting for related factors: Age, gender, education, and family income

Participants with primary glaucoma were significantly older than those without (mean age 63.0 (11.0) vs. 58.5 (5.3) years, *P* < 0.001) and were more likely to be female (*P* = 0.013), less educated (*P* < 0.001) and poorer (*P* = 0.003), (Table 1). After adjustment for age, gender, education and income, VF and QOL total score were significantly lower among participants with glaucoma (87.9 ± 15.2, 95.5 ± 12.8) compared to controls (94.4 ± 4.4,100.0 ± 0.0) (*P* = 0.015, *P* = 0.03301).

### Utility values and VF-QOL

The mean (standard deviation [SD]) UVs for glaucoma cases (n = 98/99, 99.0%) who responded to the EQ-5D was 0.98 (0.04). Among participants without primary glaucoma who completed the EQ-5D (n = 101/102, 99.0%), UVs were not significantly higher compared to participants with glaucoma (*P* = 0.092). The UVs of glaucoma cases with older age and lower family income were significantly lower than those without (*P* = 0.022, *P* = 0.039 respectively), while gender, education, self-reported systemic comorbidities, participation in New Rural Cooperative Medical Scheme (NCMS) health insurance, type of glaucoma (PACG vs. POAG), presenting visual acuity in the better-seeing eye, presence of blindness (n = 27, 39%), severity of glaucoma and awareness of diagnosis were not correlated with EQ-5D UVs. Utility values were significantly lower among participant with lower VRQOL scores (55.4 ± 11.5) compared to those with higher VRQOL scores (99.1 ± 2.8) (0.92 ± 0.08 vs. 0.99 ± 0.03, *P* = 0.036), even after adjustment for age and family income (*P* = 0.006) (Table 2). Among glaucoma participant, UVs were not significantly correlated with visual field mean defect or presenting visual acuity in the better-seeing eye, although a moderate correlation was found with the VRQOL score (*r* = 0.380, *P* < 0.001).

**Table 2:** Univariate Analysis of EQ-5D scores of Primary Glaucoma Participants Across Sociodemographic and Clinical Characteristics(n = 98)

Variable	N	Utility values Mean (Standard Deviation [SD])	P
Age, y			
≥61 y <61 y	57 41	0.98 ± 0.05 0.99 ± 0.02	**0.022**
Gender			
Male	37	0.99 ± 0.04	0.506
Female	61	0.98 ± 0.04	
Education level			
Less than primary school Primary or above	34 64	0.98 ± 0.03 0.98 ± 0.05	0.759
Family income			
<$515 (median for the area) ≥$515	40 31	0.97 ± 0.06 0.99 ± 0.02	**0.039**
Participant in rural New Cooperative Medical Service health insurance			
No	37	0.98 ± 0.04	0.309
Yes	61	0.99 ± 0.04	
Type of glaucoma			
PACG	32	0.98 ± 0.05	0.301
POAG	66	0.99 ± 0.03	
Comorbidity †			
Present	52	0.98 ± 0.05	0.068
Absent	46	0.99 ± 0.03	
Presenting visual acuity in the better-seeing eye			
<6/18	22	0.98 ± 0.04	0.559
≥6/18	76	0.98 ± 0.04	
Blind (Presenting visual acuity < 3/60) in either eye			
Yes No	27 71	0.97 ± 0.06 0.99 ± 0.03	0.077
Mean defect in the better-seeing eye			
Mild	37	0.99 ± 0.04	0.784
Moderate	15	0.98 ± 0.03	
Severe	16	0.98 ± 0.03	
Aware of diagnosis of glaucoma			
Yes	21	0.98 ± 0.06	0.474
No	77	0.98 ± 0.04	
VF total score (N, Mean ± SD)			
Normal vision group (93.4 ± 3.8)	80	0.99 ± 0.03	0.062
Impaired vision group (57.6 ± 18.0)	15	0.96 ± 0.07	
VRQOL total score (N, Mean ± SD)			
Normal vision group (99.1 ± 2.8)	88	0.99 ± 0.03	**0.036**
Impaired vision group (55.4 ± 11.5)	8	0.92 ± 0.08	

† Comorbidity present: Self-reported comorbidities include diabetes, arthritis, stroke/brain hemorrhage, high blood pressure, angina, heart attack, heart failure, and asthma

One participants with primary glaucoma not complete Visual Function scale.The VF (n = 96) and QOL (n = 97) scores were 87.9 (15.2) and 95.5 (12.8) among respondents with primary glaucoma (n = 97/99, 98.0%). A higher VF score was present among men (*P* = 0.028), those with greater income (*P* = 0.001), POAG as compared to PACG (*P* < 0.001), absence of systemic comorbidities (*P* = 0.005), PVA ≥ 6/18 in the better-seeing eye (*P* = 0.023), no blindness (*P* < 0.001) and awareness of their glaucoma diagnosis (*P* = 0.005). No correlation was found between the severity of glaucoma measured by MD and VF score (Table 3).

**Table 3:** Univariate Analysis of Visual Function Quality of Life Scores of Primary Glaucoma Participants Across Sociodemographic and Clinical Characteristics

Variable	N	Visual Function scale	P-value	N	Quality of Life scale	P-value
Age						
≥61 y <61 y	57 39	85.7 ± 18.8 91.0 ± 9.6	**0.061**	58 39	94.5 ± 13.6 96.9 ± 11.5	0.369
Gender						
Male	37	91.7 ± 10.7	**0.028**	37	96.6 ± 10.1	0.496
Female	59	85.4 ± 17.1		60	94.8 ± 14.2	
Education level						
Less than primary school Primary or above	34 62	84.9 ± 18.4 89.5 ± 13.0	0.155	34 61	95.8 ± 12.3 95.3 ± 13.1	0.847
Family income						
<$515 (Median for the area) ≥$515	40 30	82.2 ± 20.0 93.9 ± 6.8	**0.001**	40 30	92.0 ± 17.5 98.3 ± 7.7	**0.047**
Participates in rural New Cooperative Medical Service health insurance						
No	35	84.8 ± 19.5	0.196	36	90.4 ± 19.0	**0.017**
Yes	61	89.6 ± 11.9		61	98.5 ± 5.1	
Type of glaucoma						
PACG	32	77.8 ± 21.4	**< 0.001**	32	89.0 ± 18.1	**< 0.001**
POAG	64	92.9 ± 6.8		63	98.7 ± 7.5	
Self-reported comorbidity †						
Present	50	85.2 ± 17.5	**0.005**	51	94.7 ± 14.4	0.074
Absent	46	90.7 ± 11.8		46	96.4 ± 10.8	
Presenting visual acuity in the better-seeing eye						
<6/18	21	77.9 ± 23.4	**0.023**	21	87.9 ± 21.0	0.052
≥6/18	75	90.6 ± 10.6		76	97.6 ± 8.4	
Blind (Presenting visual acuity < 3/60) in either eye						
Yes No	27 69	75.1 ± 22.6 92.8 ± 6.2	**< 0.001**	27 70	85.1 ± 21.0 99.5 ± 1.7	**0.001**
Mean defect in the better-seeing eye						
Mild	36	91.7 ± 6.8	0.070	36	98.2 ± 5.9	0.245
Moderate	14	86.5 ± 12.8		14	93.4 ± 17.5	
Severe	16	82.6 ± 22.3		16	92.6 ± 17.9	
Aware of glaucoma						
Yes No	21 75	77.0 ± 19.4 90.9 ± 12.3	**0.005**	21 76	89.4 ± 18.2 97.2 ± 10.4	0.074

† Comorbidity present: Self-reported comorbidities included diabetes, arthritis, stroke/brain hemorrhage, high blood pressure, angina, heart attack, heart failure, and asthma

Among persons with glaucoma, a higher QOL score was present among those with greater family income (*P* = 0.047), POAG vs. PACG (*P* < 0.001), without blindness (*P* = 0.001), and participants in rural New Cooperative Medical Service health insurance (*P* = 0.017) (Table 3). Age, gender, education, severity of glaucoma, and self-reported comorbidities were not associated with VRQOL score.

In multiple linear regression models, UV among glaucoma cases was associated with VRQOL total score (*β* = 2.791, *P* = 0.007) (Table 4) and significantly related to the dimensions of self-care (*β* = 0.844, *P* < 0.001) and mobility (*β* = -0.450, *P* = 0.026). VF score among participanst with glaucoma was associated with presenting visual acuity in the better-seeing eye (6/18) (*β* = 0.247, *P* = 0.027) and a diagnosis of POAG vs. PACG (*β* = -0.313, *P* = 0.013). VRQOL score was associated with participation in New Cooperative Medical Service health insurance (*β* = 0.265, *P* = 0.020) and POAG (*β* = -0.274, *P* = 0.015) (Table 4).

**Table 4:** Multivariate analysis of the relationships between potential predictive fctors and EQ-5D/VF-QOL in glaucoma participants

Independent variables	Model 1 (EQ-5D, n = 71)		Model2 (VF score, n = 70)		Model3 (QOL score, n = 70)	
	β	P	β	P	β	P
Age	-0.168	0.190	-	-	-	-
Gender	-	-	-0.148	0.167	-	-
VRQOL total score	**2.791**	**0.007**	-	-	-	-
Family income	0.098	0.761	0.108	0.334	0.082	0.467
PACG vs. POAG	-	-	**-0.313**	**0.013**	**-0.274**	**0.015**
PVA in the better-seeing eye > 6/18	-	-	**0.247**	**0.027**	0.188	0.111
Participation in New Cooperative Medical Service health insurance	-	-	-	-	**0.265**	**0.020**
Self-reported comorbidity	-	-	-0.037	0.731	-	-
Aware of glaucoma	-	-	-0.168	0.164	-	-

## Discussion

To our knowledge, this is the first population study using EQ-5D to compare UVs between participants with and without glaucoma in China. Our study observed a consistent reduction in both visual function and quality of life among rural Chinese individuals living with glaucoma. Specifically, we found that primary angle-closure glaucoma participants experienced greater reductions in visual function and QOL than those with primary open-angle glaucoma.

In this population-based sample of persons with glaucoma, with or without comorbidities, UVs were substantially higher than reported in several previous studies in other countries. For instance, mean UVs of 0.76, 0.80, 0.65, and 0.89 have been reported for glaucoma participants in the United Kingdom, Sweden, Europe, and Korea, respectively [21–23]. This discrepancy may be attributed to the population-based nature of our study, while these other investigations focused on clinic-based samples. Moreover, we found that the visual function score of participants aware of their glaucoma was significantly lower than those who were unaware. It is well-established that persons presenting for glaucoma care often have more severe disease than those who do not seek care, as previously reported in China [24].

In the current study, UVs based on EQ-5D showed a weak correlation with the severity of visual field loss in the better-seeing eye, consistent with other studies which found no strong association between UVs and severity of field loss. However, previous publications have reported significantly higher UVs with mild compared to more severe field loss [23, 25], which was not observed in our study, possibly due to the smaller number of participants and milder damage in this population-based study. Although UVs were not significantly correlated with the visual field, a moderate correlation was found with the VRQOL total score (r = 0.380, P < 0.001). Glaucoma participants with lower VRQOL total scores had poorer HRQOL as reflected in their UVs. The current study found that participants within the lower VRQOL total score subgroup had significantly lower UV levels, suggesting that preserving the quality of vision in these participants may be cost-effective.

Our findings may be useful for cost-utility analysis(CUA) for interventions of glaucoma participants [5]. It has also been recommended that mapping the VRQOL- related scale to the EQ-5D, is more suitable for use in an ophthalmology clinic [26].

With limited medical resources in rural China, older women with low income and lower education may be appropriate targets for glaucoma outreach efforts, which recent modeling has suggested may be cost-effective [5]. In the current study, participants with glaucoma were older and more likely to be female and poor. Besides, the visual function of older and poorer participants with glaucoma was poor. An association with female hormones may explain this female propensity for glaucoma and the associated loss of visual function [27–29]. The Collaborative Normal-Tension Glaucoma Study (CNTGS) [30] reported that the probability of progression of normal-tension glaucoma among women was 1.85-fold higher than among men.

Other studies have observed an association between poverty and low education [31–33] with health-seeking behavior and compliance. Follow-up [34] and treatment compliance are crucial to controlling chronic diseases such as glaucoma, which may explain our findings. Indeed, health education for this patient population is important.

More attention should be paid to interventions targeting angle-closure glaucoma. We found that VF and QOL were worse among participants with PACG compared to POAG, consistent with findings of higher blindness risk at presentation in the former group in China [35].

To improve access to healthcare for rural Chinese participants with glaucoma, it is necessary to leverage the NCMS rural health insurance system. Participation in NCMS has been reported to enhance access to eye care services such as cataract surgery by reducing out-of-pocket costs [36]. Importantly, a significantly higher visual-related quality of life score was observed among participants covered by rural cooperative medical services in the current study compared to those without.

Comorbidities should be considered when screening or management is undertaken for glaucoma [36]. The presence of complications is not only related to the development of glaucoma but also significantly reduces the visual function of participants. Diabetes [37], duration of diabetes, and fasting blood glucose levels have been associated with a significantly increased risk for glaucoma and a mild increase in intraocular pressure [38]. Additionally, hypertension [39, 40], and impaired glucose tolerance reportedly increase the risk for normal-tension glaucoma, suggesting that metabolic syndrome may play a role in the pathogenesis of glaucoma.

Our data suggest that intervention and management of comorbidities may help improve glaucoma participants’ visual function. The strengths of our study included the population-based design and high rates of response on most questions. While the ongoing collection of longitudinal data for the HES is expected to address limitations associated with the use of cross-sectional data in our study, we acknowledge that the modest size of our glaucoma patient cohort may limit the generalizability of our findings. Nonetheless, the HES is the largest population survey of eye disease in China that has used the EQ-5D instrument, previously validated among the Chinese population. We hope that such unique data will strengthen glaucoma management in a cost-effective way and improve quality of life among rural-dwelling Chinese persons with glaucoma.

## Conclusion

Compared to age-matched population controls, participants with glaucoma had worse VF and VR-QOL score. Comparatively to those with POAG, participants with PACG had worse VF and VR-QOL score. In contrast to glaucoma participants who had a normal VR-QOL score, those glaucoma participants who had impaired VR-QOL score showed a significantly decreased UVs. It suggested that any early intervention measure may be potentially cost-effective for glaucoma, especially for PACG. UVs could be utilized to enhance glaucoma public health initiatives and cost-effectiveness research studies in rural China.

### Electronic supplementary material

Below is the link to the electronic supplementary material.

Supplementary Material 1

## Data Availability

The datasets generated and analyzed during the current study are not publicly available but are available from the corresponding author upon reasonable request.

## Abbreviations

HRQOL: Health-related quality of life
HES: Handan Eye study
POAG: Primary open-angle glaucoma
PACG: Primary angle-closure glaucoma
VF - QOL: Visual function – quality of life
VR – QOL: Visual - related quality of life
UVs: Utility values
GDP: Gross domestic product
RNFL: Retinal nerve fiber layer
CEA: Cost-effectiveness analysis
QALYs: Quality adjusted life years
NICE: National Health institute for clinical excellence
VFL: Visual field loss
PVA: Present visual acuity
MD: Mean defect
NCMS: New Rural Cooperative Medical Scheme
CNTGS: Collaborative Normal-Tension Glaucoma Study
