# Supplementary material for: Impact of primary glaucoma on health-related quality of life in China: the handan eye study
Source: BMC Ophthalmol. 2023 Sep 14;23:377. doi: 10.1186/s12886-023-03106-w (PMC10503180; doi:10.1186/s12886-023-03106-w)
Supplement: Supplementary file 1 — Supplementary Material 1 [file 12886_2023_3106_MOESM1_ESM.docx]

**Impact of primary glaucoma on Health-Related Quality of Life in China: The Handan Eye Study**

Di Song^2^ MD, Sujie Fan^5^ MD, Qiang Zhou^6^ MD, Xiaohui Yang^8^ MD, Sizhen Li^4,8^ MD, Lynne Lohfeld^7^ Ph.D., Weihe Zhou^1,3^ Ph.D., Nathan Congdon^7,9^ MD, MPH., *Yuanbo Liang^1,3^ MD, Ph.D., *Ningli Wang^8^ MD, Ph.D.

1 Clinical & Epidemiological Eye Research Center, The Affiliated Eye Hospital of Wenzhou Medical University. Wenzhou, Zhejiang, China

2 The First People's Hospital of Huzhou, The First Affiliated Hospital of Huzhou Teacher College, Huzhou, China.

3 Glaucoma Institute, Wenzhou Medical University, Wenzhou, Zhejiang, China

4 Department of Ophthalmology and Visual Sciences, The Chinese University of Hong Kong, Hong Kong, China

5 Handan Eye Hospital, Handan, China

6 Beijing Chaoyang Hospital, Beijing, China

7 Centre for Public Health, Queen' s University, Belfast, UK

8 Beijing Tongren Eye Center, Beijing Tongren Hospital, Capital Medical University; Beijing Ophthalmology & Visual Science Key Lab, Beijing, China

9 Orbis International, New York, USA.

* Corresponding authors.

Yuanbo Liang

Address: No. 270, Xue Yuan Xi Road, Wenzhou, Zhejiang, China. 325000.

Email address: [yuanboliang@126.com](about:blank)

Ningli Wang

Address: Beijing Tongren Eye Center, Beijing Tongren Hospital, Capital Medical University; Beijing Ophthalmology & Visual Science Key Lab, Beijing, China.

Email address: [wningli@163.vip.com](mailto:wningli@163.vip.com)

**Patient and Public Involvement：**

A letter was sent to each potential subject's home address inviting him/her to participate in the study. At least three days before, a visit to the potential subject's home was made by local organizers (usually the head of the village and/or village doctors), and the nature of the study was explained in detail by recruitment staff (one ophthalmologist and two assistants). The subject was invited for an eye examination, and travel to the clinic was scheduled. All conversations conducted by HES staff were carried out in the local dialect. If the residents were unwilling to visit the clinic after three home visits, a slightly abbreviated examination conducted in the village (as opposed to the designated examination center) was offered. A brief home examination was offered for those who did not attend the village examination. Persons who had moved from the given home address, had not lived in the village in the past 6 months, were deceased or terminally ill (life expectancy less than 3 months, decided by the village doctors), or had severe psychiatric disease were ineligible^1^.

**The definition of PACG And POAG**

PACG was defined as a. if either eye had the posterior trabecular meshwork not visible for 180° or more on gonioscopy; b. if there was evidence of peripheral anterior synechiae (PAS) and/or IOP ≥21 mm Hg in the eye; c. evidence of glaucomatous damage to the optic nerve. POAG was defined as: a. has no secondary cause of glaucoma and open angles on gonioscopy. b. evidence of glaucomatous damage of the optic nerve

**References**

1.Liang YB, Friedman DS, Wong TY, et al. Rationale, design, methodology, and baseline data of a population-based study in rural China: the Handan Eye Study. Ophthalmic Epidemiol 2009;16(2):115-27.
